# Supplementary material for: Multiplets in scRNA-seq data: Extent of the problem and efficacy of methods for removal
Source: PLoS One. 2025 Oct 30;20(10):e0333687. doi: 10.1371/journal.pone.0333687 (PMC12574873; doi:10.1371/journal.pone.0333687)
Supplement: S1 Table — (PDF) [file pone.0333687.s001.pdf]

S1 Table Overview of dataset information

Dataset	Source	Tissue / Samples	Library prep chemistry	Multiplexing method	Sequencing platform	Droplets	Multiplets	Multiplet Rate (%)
pbmc-ch	GEO GSE108313 [1]	Human PBMCs (8 donors)	10x Genomics Chromium Single Cell 3' v2	Antibody HTO	Illumina HiSeq 2500	15,272	2,545	16.66
cline-ch	GEO GSE108313 [1]	Human cell lines: HEK293T, K562, KG1, THP1	10x Genomics Chromium Single Cell 3' v2	Antibody HTO	Illumina HiSeq 2500	7,954	1,465	18.42
mkidney	GEO GSE140262 [2]	Mouse kidney cells (2 samples)	10x Genomics Chromium Single Cell 3' v3	Lipid CMO (MULTI-seq)	Illumina HiSeq 4000	21,179	7,901	37.31
gold standard	10x Genomics [3]	Human PBMCs (donors 1–2) from healthy individuals; BMMCs (donor 3) and PBMCs (donor 4) from ALL patients*	10x Genomics 5' Immune Profiling	Antibody HTO (TotalSeq-C)	Illumina NovaSeq 6000	27,504	7,186	26.13

Notes:
*For the gold standard dataset, droplets from donors with ALL (donors 3 and 4) were excluded from the analysis.
PBMCs = Peripheral Blood Mononuclear Cells.
BMMCs = Bone Marrow Mononuclear Cells.
HTO = Hashtag oligonucleotides (antibody-derived barcodes) used for sample multiplexing (cell hashing).
CMO = Cholesterol-modified oligonucleotides (lipid-tagged barcodes) used for sample multiplexing (cell hashing).
TotalSeq-C = Commercial antibody–oligo conjugate product line (BioLegend) used for sample multiplexing (cell hashing).
MULTI-seq = Published lipid-oligo based method used for sample multiplexing (cell hashing).

References

[1] Stoeckius M, Zheng S, Houck-Loomis B, Hao S, Yeung BZ, Mauck WM, et al. Cell Hashing with barcoded antibodies enables multiplexing and doublet detection for single cell genomics. Genome Biology. 2018;19:1–12. doi:10.1186/S13059-018-1603-1/FIGURES/3.

[2] Bernstein NJ, Fong NL, Lam I, Roy MA, Hendrickson DG, Kelley DR. Brief Report Solo : Doublet Identification in Single-Cell RNA-Seq via Semi-Supervised Deep Learning. Cell Systems. 2020;11:1–7. doi:10.1016/j.cels.2020.05.010.

[3] 10x Genomics. Demultiplexing and Analyzing 5' Immune Profiling Libraries Pooled with Hashtags - 10x Genomics;. Available from: https://www.10xgenomics.com/analysis-guides/demultiplexing-and-analyzing-5%E2%80%99-immune-profiling-libraries-pooled-with-hashtags.
