# Supplementary figures and images for: Multiplets in scRNA-seq data: Extent of the problem and efficacy of methods for removal
Source: PLoS One. 2025 Oct 30;20(10):e0333687. doi: 10.1371/journal.pone.0333687 (PMC12574873; doi:10.1371/journal.pone.0333687)

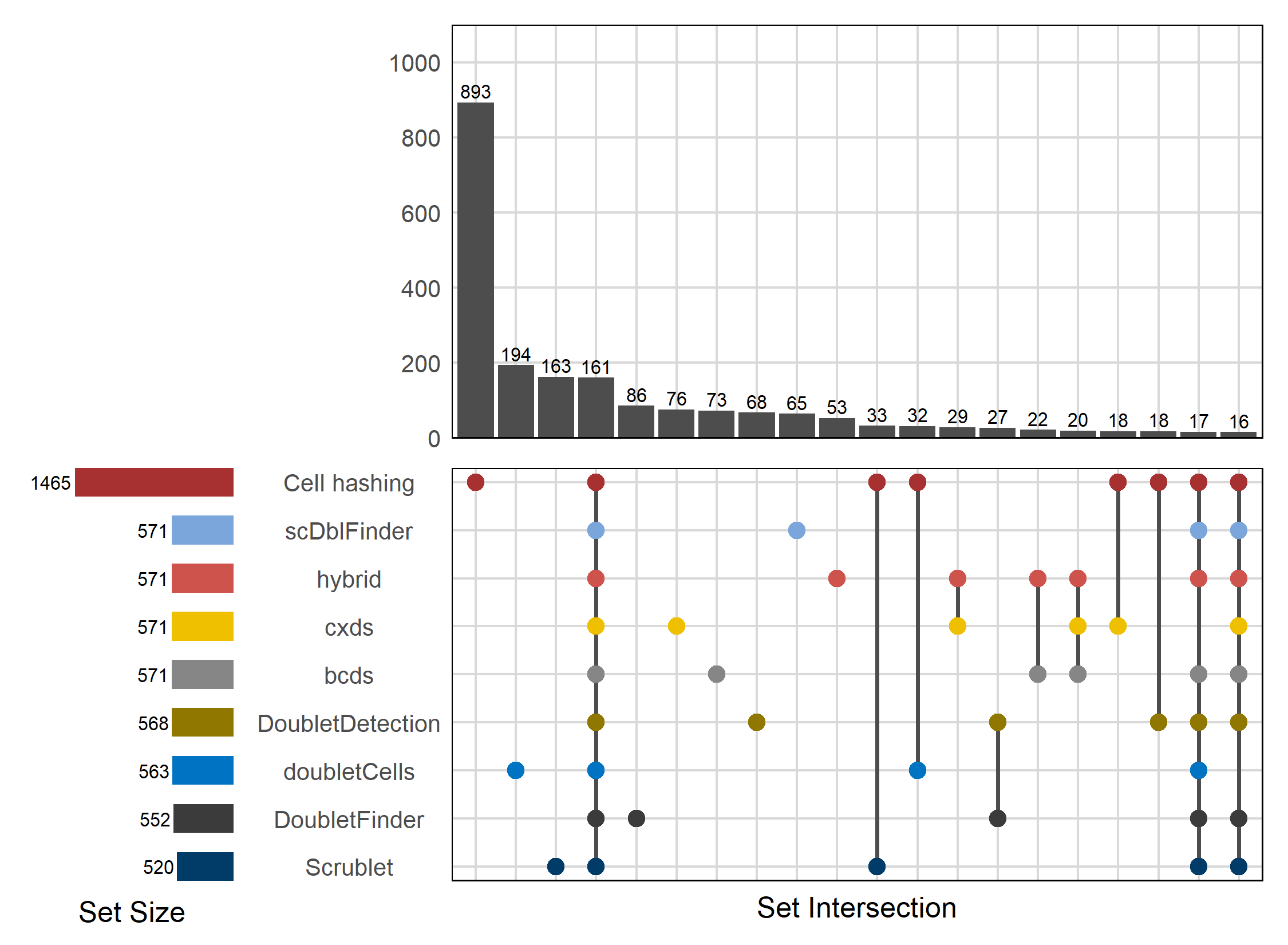

Supplement: S1 Fig — UpSet plot showing sets and set intersections of multiplets identified by the tools and by cell hashing in the cline dataset. Horizontal bars (bottom left) show the total number of multiplets identified by each method. Vertical bars (top right) show the sizes of the sets of detected multiplets. If a point is present for a method underneath a bar (bottom right plot panel), this means that this method has identified the particular multiplets that are members of the set denoted by that bar. If multiplet points are present underneath a bar, this means that multiple methods identified the same set of multiplets, i.e. their sets of multiplets intersect. Points are colored by method. Only the 20 largest multiplet sets and set intersections are shown. Note the y axis break. (TIF) [file pone.0333687.s002.tif]

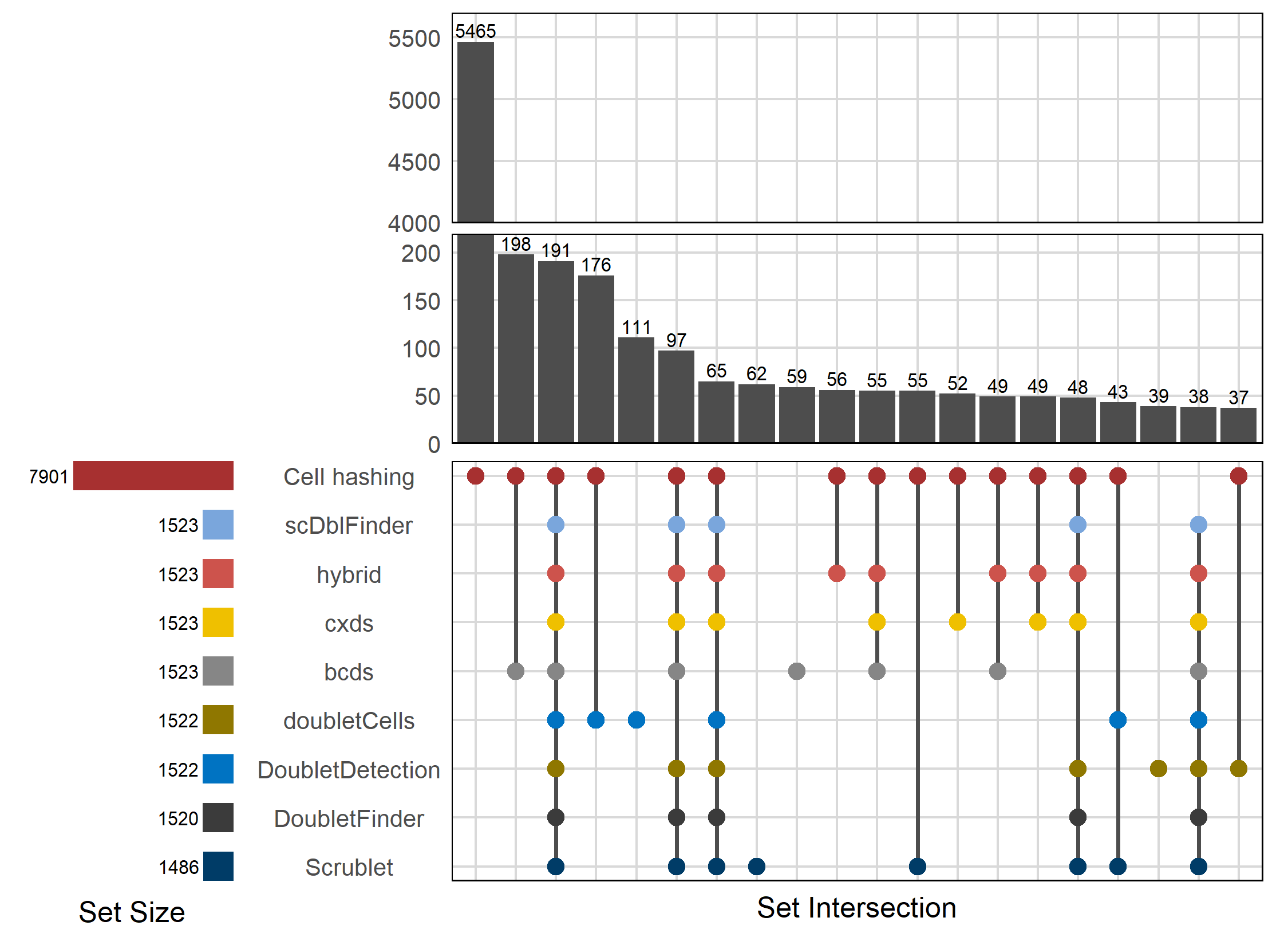

Supplement: S2 Fig — UpSet plot showing sets and set intersections of multiplets identified by the tools and by cell hashing in the mkidney dataset. Horizontal bars (bottom left) show the total number of multiplets identified by each method. Vertical bars (top right) show the sizes of the sets of detected multiplets. If a point is present for a method underneath a bar (bottom right plot panel), this means that this method has identified the particular multiplets that are members of the set denoted by that bar. If multiplet points are present underneath a bar, this means that multiple methods identified the same set of multiplets, i.e. their sets of multiplets intersect. Points are colored by method. Only the 20 largest multiplet sets and set intersections are shown. Note the y axis break. (TIF) [file pone.0333687.s003.tif]

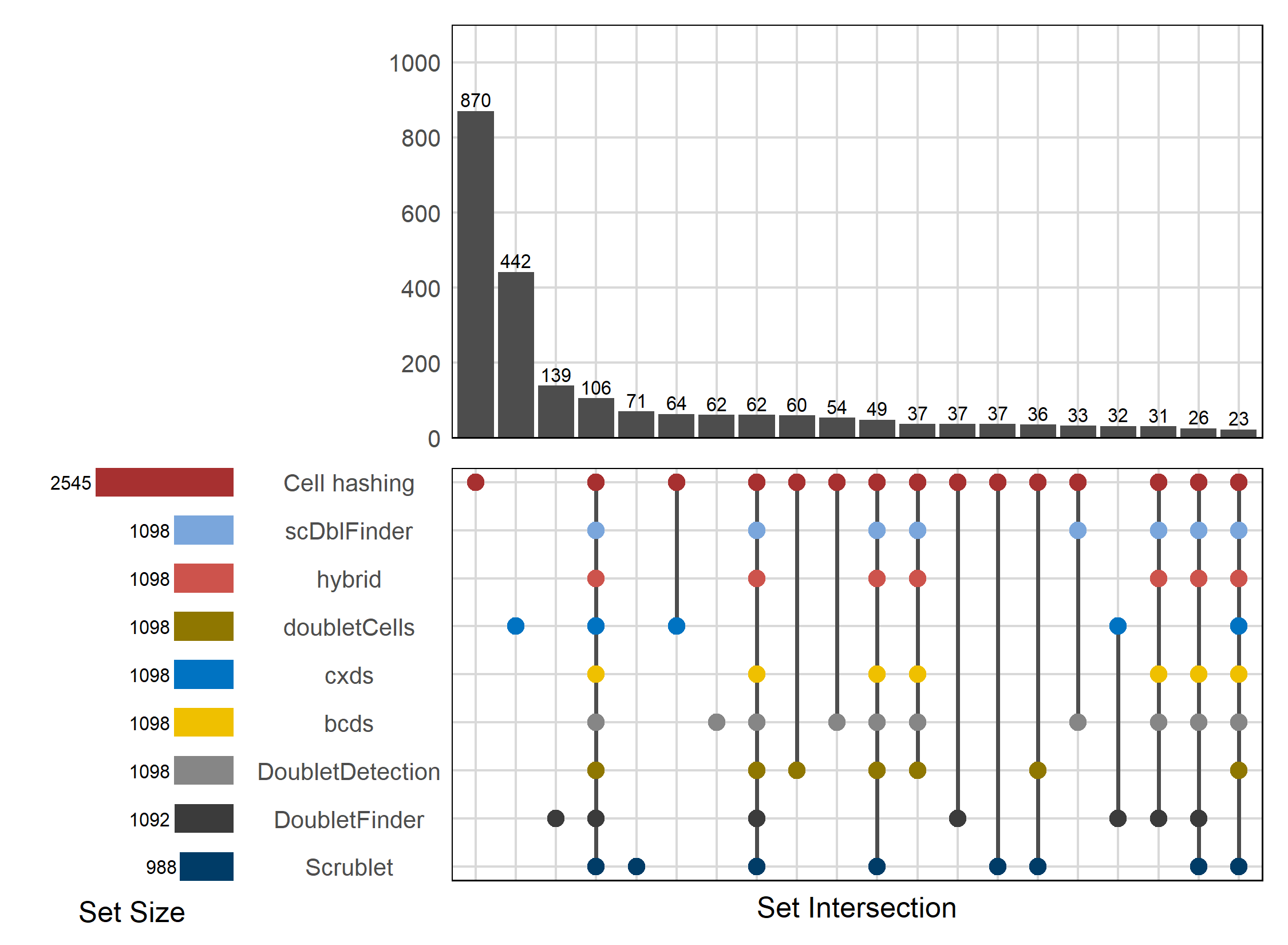

Supplement: S3 Fig — UpSet plot showing sets and set intersections of multiplets identified by the tools and by cell hashing in the pbmc dataset. Horizontal bars (bottom left) show the total number of multiplets identified by each method. Vertical bars (top right) show the sizes of the sets of detected multiplets. If a point is present for a method underneath a bar (bottom right plot panel), this means that this method has identified the particular multiplets that are members of the set denoted by that bar. If multiplet points are present underneath a bar, this means that multiple methods identified the same set of multiplets, i.e. their sets of multiplets intersect. Points are colored by method. Only the 20 largest multiplet sets and set intersections are shown. Note the y axis break. (TIF) [file pone.0333687.s004.tif]

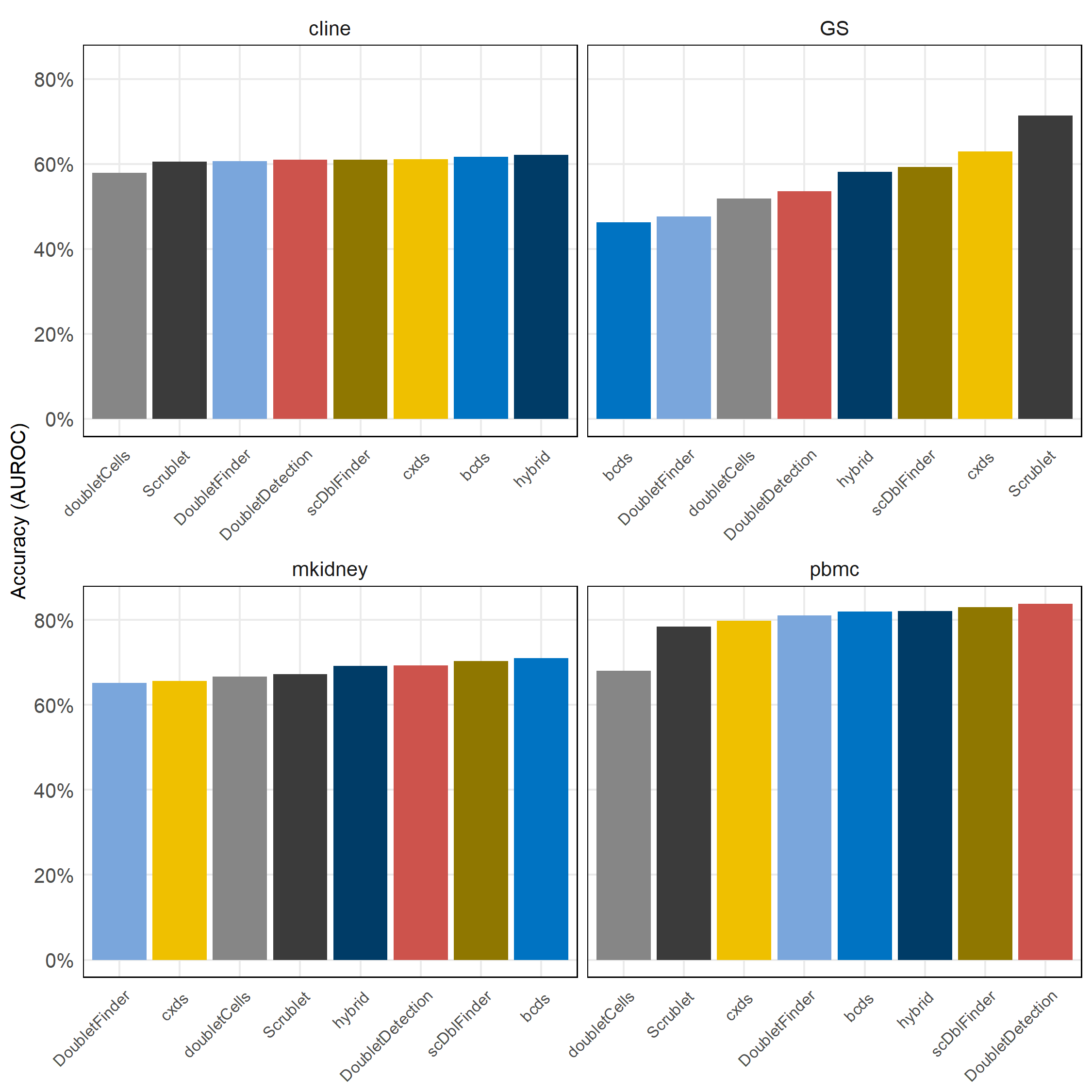

Supplement: S4 Fig — (TIF) [file pone.0333687.s005.tif]

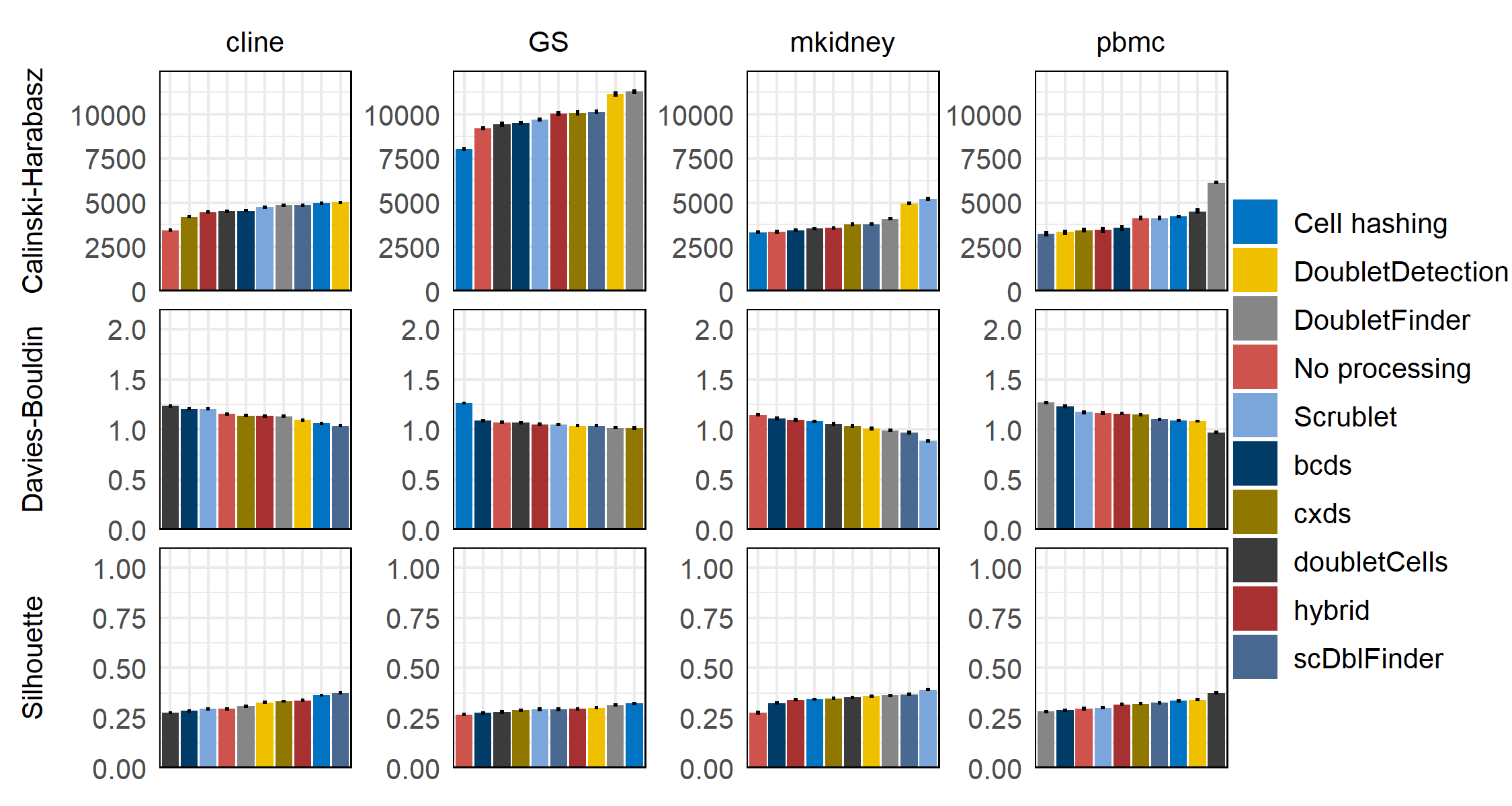

Supplement: S5 Fig — Bar plot of clustering quality after the removal of multiplets detected by each of the eight methods tested and by cell hashing. Clustering was evaluated using three complementary metrics: the Calinski–Harabasz Index (higher is better; rewards global separation and a higher number of compact clusters), the Davies–Bouldin Index (lower is better; rewards compact clusters that are well-separated from their nearest neighbors), and the Silhouette value (closer to 1 is better; rewards droplets being closer to their own cluster than to other clusters). To account for differences in dataset size, we fixed the number of droplets per dataset to a common value—rounded down to just below the smallest dataset—and performed stratified subsampling, repeating this procedure 100 times per method and metric. Bars show the mean clustering quality, and error bars denote variability across subsamples. (TIF) [file pone.0333687.s006.tif]

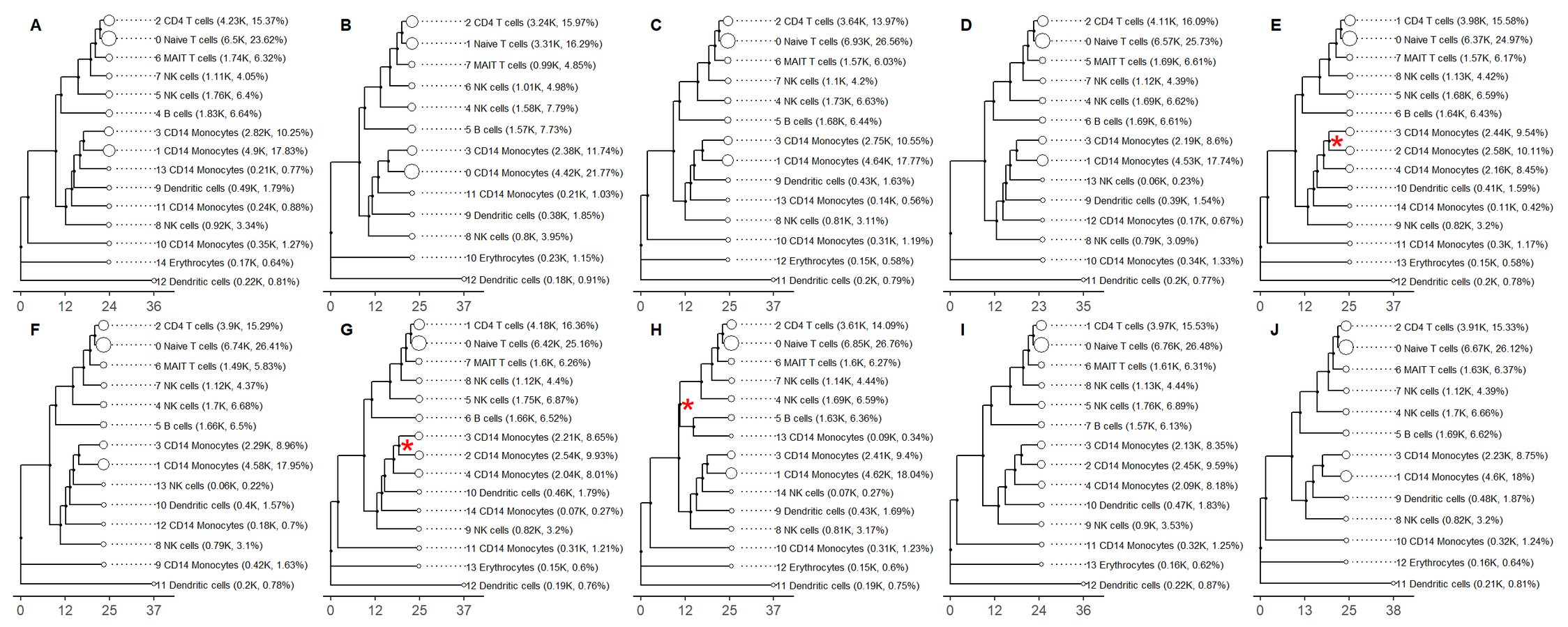

Supplement: S6 Fig — (A) Gold standard dataset clustered with scBubbletree without removing multiplets. (B) Gold standard dataset clustered with scBubbletree after removing cell-hashing multiplets. (C) Gold standard dataset clustered with scBubbletree after removing DoubletCells multiplets. (D) Gold standard dataset clustered with scBubbletree after removing cxds multiplets. (E) Gold standard dataset clustered with scBubbletree after removing bcds multiplets. (F) Gold standard dataset clustered with scBubbletree after removing hybrid multiplets. (G) Gold standard dataset clustered with scBubbletree after removing scDblFinder multiplets. (H) Gold standard dataset clustered with scBubbletree after removing Scrublet multiplets. (I) Gold standard dataset clustered with scBubbletree after removing DoubletDetection multiplets. (J) Gold standard dataset clustered with scBubbletree after removing DoubletFinder multiplets. The robustness of clustering was assessed with bootstrapping, i.e. by the number of times a cluster emerged out of 1000 bootstrap resamples of the data during clustering. Red asterisks denote the only branches with a bootstrapping value less than 1000. (TIF) [file pone.0333687.s007.tif]
